# Electrocatalytic reduction of carbon dioxide to carbon monoxide and methane at an immobilized cobalt protoporphyrin

**DOI:** 10.1038/ncomms9177

**Published:** 2015-09-01

**Authors:** Jing Shen, Ruud Kortlever, Recep Kas, Yuvraj Y. Birdja, Oscar Diaz-Morales, Youngkook Kwon, Isis Ledezma-Yanez, Klaas Jan P. Schouten, Guido Mul, Marc T. M. Koper

**Affiliations:** 1 Leiden Institute of Chemistry, Leiden University, PO Box 9502, 2300 RA Leiden, The Netherlands; 2 PhotoCatalytic Synthesis Group, MESA+ Institute for Nanotechnology, Faculty of Science and Technology, University of Twente, Meander 229, PO Box 217, 7500 AE Enschede, The Netherlands

## Abstract

The electrochemical conversion of carbon dioxide and water into useful products is a major challenge in facilitating a closed carbon cycle. Here we report a cobalt protoporphyrin immobilized on a pyrolytic graphite electrode that reduces carbon dioxide in an aqueous acidic solution at relatively low overpotential (0.5 V), with an efficiency and selectivity comparable to the best porphyrin-based electrocatalyst in the literature. While carbon monoxide is the main reduction product, we also observe methane as by-product. The results of our detailed pH-dependent studies are explained consistently by a mechanism in which carbon dioxide is activated by the cobalt protoporphyrin through the stabilization of a radical intermediate, which acts as Brønsted base. The basic character of this intermediate explains how the carbon dioxide reduction circumvents a concerted proton–electron transfer mechanism, in contrast to hydrogen evolution. Our results and their mechanistic interpretations suggest strategies for designing improved catalysts.

## Abstract (web summary)

The conversion of carbon dioxide to useful products is a major challenge in energy research. Here, the authors report a cobalt protoporphyrin immobilized on graphite that is capable of the selective and efficient electrochemical reduction of carbon dioxide, primarily to carbon monoxide, in acidic media.

## Graphical abstract

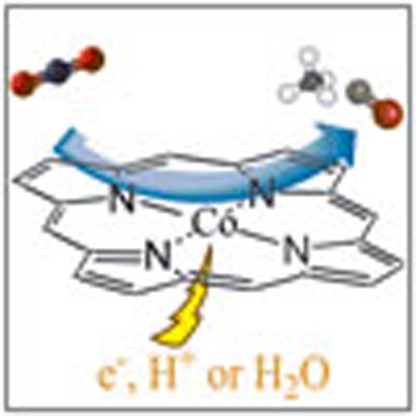

The efficient electrochemical reduction of carbon dioxide to a fuel with a high-energy density would be a major step forward in the introduction of a CO_2_ neutral energy cycle, as it would allow for the direct low-temperature conversion of photo-generated electrical current to stored chemical energy, in a manner very similar to the way nature stores solar energy. Plants fix CO_2_ from the atmosphere by photosynthesis in an enzymatic complex called Rubisco, which selectively binds CO_2_ and inserts it into existing carbon chains by reductive carboxylation. The high-energy electrons necessary for this process are photo-generated by photosystem II.

Synthetic catalysts for the electrocatalytic reduction of CO_2_, which could facilitate such an artificial CO_2_ neutral redox cycle, have been studied for many decades^1^,^2^,^3^,^4^. A main challenge in electrochemical CO_2_ reduction is to develop catalysts that are capable of reducing CO_2_ beyond the two-electron products carbon monoxide (CO), formic acid (HCOOH), and oxalate (C_2_O_4_^2−^). Unfortunately, the formation of reduction products requiring four or more electrons is invariably associated with considerable overpotentials due to the multiple intermediates involved in the reaction mechanisms^5^ (although more reduced products often have higher stability and correspondingly more positive equilibrium potentials). Metallic copper is unique in producing significant amounts of high-energy multi-electron transfer products such as methane, ethylene and ethanol^3^,^6^,^7^. Molecular catalysts that are capable of reducing CO_2_ to a product different from one of the above-mentioned two-electron products are much less common and typically involve a strong interaction with the working electrode^8^. A second important challenge in CO_2_ electrocatalysis concerns the suppression of the concomitant evolution of hydrogen, which is a dominant side reaction for CO_2_ reduction from aqueous electrolytes. Strategies for suppressing hydrogen evolution typically involve working with high(er) CO_2_ to proton ratios, such as high CO_2_ pressures or solvents with a higher CO_2_ solubility.

Recent fundamental and theoretical work has reconsidered porphyrin-based molecular catalysts for electrochemical CO_2_ reduction. Tripkovic *et al*.^9^ have performed extensive density functional theory calculations of metal-functionalized porphyrin-like graphene surfaces, and predicted the potential formation of methane and methanol from CO_2_. Costentin *et al*.^10^ considered ligand modifications of iron-based porphyrins and found that local proton sources built into the porphyrin ring give rise to high activity and good Faradaic efficiency (FE) for the reduction of CO_2_ to CO in a mixed DMF–water solvent. In fact, it has been known since the early 1980s that cobalt (Co)-based macrocyclic complexes, either in solution or adsorbed onto carbon electrodes, act as effective electrocatalysts for CO_2_ reduction, producing CO, HCOOH, methanol and methane, although at relatively high overpotential and with varying selectivity^11^,^12^,^13^,^14^,^15^.

Herein, we report on the electrochemical reduction of CO_2_ to CO and methane, as well as smaller amounts of HCOOH and methanol, on a simple Co protoporphyrin molecular catalyst immobilized onto a pyrolytic graphite (PG) electrode in a purely aqueous electrolyte solution. Previous similar work using immobilized Co porphyrins or Co phthalocyanines has shown the capability of Co-based catalysts to achieve a high FE towards CO, which is highly sensitive to pH and potential^16^,^17^,^18^. Our work confirms that immobilized Co-based porphyrins are good CO_2_ reduction electrocatalysts capable of producing multi-electron products such as methane and methanol. More significantly, our work underscores the important role of pH in steering the catalytic activity and selectivity towards CO and CH_4_, especially in the very narrow pH=1–3 range in the absence of coordinating anions. This high sensitivity to pH is explained by a mechanism highlighting the important role of the initial electron transfer in activating CO_2_ electrochemically. We also demonstrate how such a mechanism for CO_2_ reduction manifests experimentally and how this property can be exploited to suppress concomitant hydrogen evolution. Furthermore, we show that the overpotential and corresponding turnover frequency (TOF) for CO_2_ reduction of our catalyst compare favourably to the best molecular porphyrin-based catalyst in the literature^10^. Therefore, we believe that these insights may have significant implications for the design of new and improved molecular catalyst electrodes and for the formulation of optimized process conditions for efficient electrochemical CO_2_ reduction to CO as well as to products reduced to a more significant degree.

## Results

### Voltammetry and online electrochemical mass spectrometry

The Co protoporphyrin-coated PG (CoPP-PG) electrode was prepared following a procedure described earlier^19^ and was detailed in the Methods section. *In situ* electrochemical scanning tunnelling microscopy and atomic force microscopy images of iron and zinc protoporphyrins on basal plane graphite electrodes by Tao *et al*.^20^ suggest that these molecules form monolayer films on the electrode with the molecules lying flat. The blank cyclic voltammograms of the PG electrode, the CoPP-PG electrode in 0.1 M HClO_4_ and the voltammetry of the dissolved CoPP in the same electrolyte are compared in Supplementary Fig. 1. The voltammetry in Supplementary Fig. 1 shows the reversible redox peak of the Co^3+^/Co^2+^ transition at 0.8–0.85 V versus reversible hydrogen electrode (RHE), from which the coverage of the Co-PP on the PG electrode can be determined to be ca. 4 × 10^−10^ mol cm^−2^, which is in good agreement with previous experiments of protoporphyrins on PG^19^,^21^. No further redox transition of the CoPP is observed at more negative potential, with the onset of hydrogen evolution being at ca. −0.5 V_RHE_. However, we note that we have previously observed a Co^2+^/Co^+^ transition at ca. −0.6 V versus NHE for CoPP immobilized in a DDAB (didodecyl dimethylammonium bromide) film on PG^19^. The observation of this peak in the DDAB films may be related to the higher hydrophobicity of DDAB. The Co^2+^/Co^+^ redox transition has previously been associated with the onset of electrocatalytic hydrogen evolution on Co porphyrins^22^.

**Figure 1:**
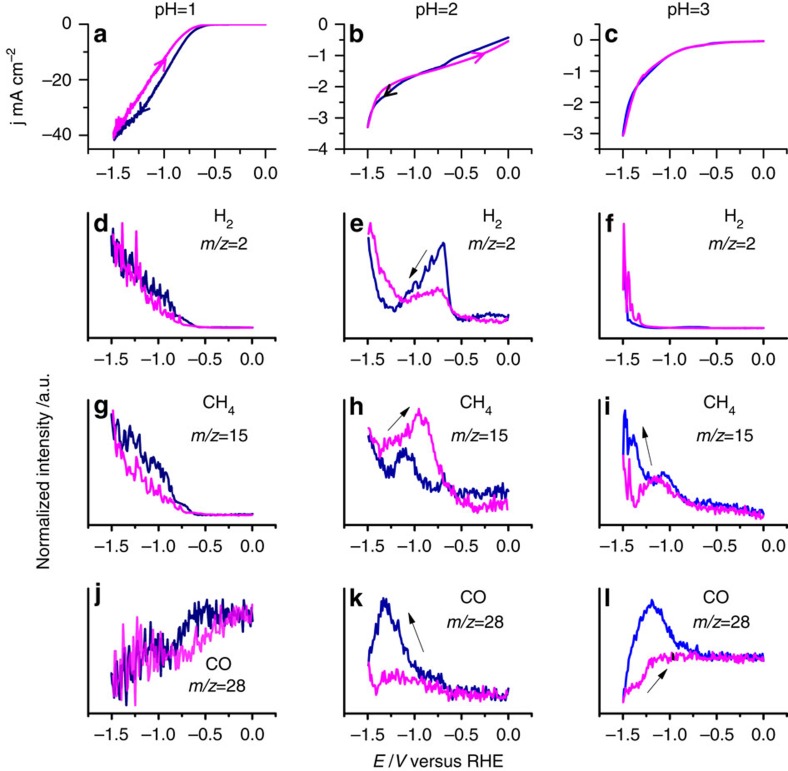
Voltammetry and volatile product identification by online electrochemical mass spectrometry. This figure shows the electrochemical reduction of CO_2_ on Co protoporphyrin immobilized on a PG electrode and the various volatile products detected by OLEMS. (**a**) CV in 0.1 M HClO_4_; (**b**) CV in 10 mM HClO_4_+90 mM NaClO_4_; (**c**) CV in 1 mM HClO_4_+99 mM NaClO_4_; (**d**) *m/z*=2 (H_2_) signal in 0.1 M HClO_4_; (**e**) *m/z*=2 (H_2_) signal in 10 mM HClO_4_+90 mM NaClO_4_; (**f**) *m/z*=2 (H_2_) signal in 1 mM HClO_4_+99 mM NaClO_4_; (**g**) *m/z*=15 (CH_4_) signal in 0.1 M HClO_4_; (**h**) *m/z*=15 (CH_4_) signal in 10 mM HClO_4_+90 mM NaClO_4_; (**i**) *m/z*=15 (CH_4_) signal in 1 mM HClO_4_+99 mM NaClO_4_; (**j**) *m/z*=28 (CO) signal in 0.1 M HClO_4_; (**k**) *m/z*=28 (CO) signal in 10 mM HClO_4_+90 mM NaClO_4_; (**l**) *m/z*=28 (CO) signal in 1 mM HClO_4_+99 mM NaClO_4_. Scan rate was in all cases 1 mV s^−1^. Blue lines are negative-going (forward) scans; magenta lines are positive-going (return) scans. Supplementary Fig. 4 shows the same data with the unnormalized MS signals, as well as the signals obtained in the first and second CV scan.

Figure 1 shows the voltammetry at 1 mV s^−1^ of the CoPP-PG electrode in unbuffered 0.1 M perchlorate solution of pH=1–3, saturated with CO_2_, together with the mass signals corresponding to H_2_ (*m/z*=2), CH_4_ (*m/z*=15, corresponding to the CH_3_ fragment) and CO (m/z=28) as measured simultaneously using online electrochemical mass spectrometry (OLEMS)^23^. The OLEMS experiment samples the gases formed at the electrode surface by a tip covered with a hydrophobic membrane placed at a distance of ca. 10 μm from the surface. This technique can follow gas production online during cyclic voltammetry (CV). Calibration of our experiment is cumbersome as the signals depend on parameters that are not easy to control (tip distance and tip porosity). Quantitative measurements were therefore performed using long-term electrolysis combined with gas chromatography (to be discussed later). Depending on the quality of the gas-sensing tip used in the OLEMS experiment shown in Fig. 1, *m/z*=31 was also measured, corresponding to the formation of methanol (Supplementary Fig. 2). Using high-performance liquid chromatography (HPLC), we could also detect HCOOH as one of the products (Supplementary Fig. 3), although both HCOOH and methanol appear to be minority products under these conditions. This confirms, for the first time in a single study, that all four products, CO, HCOOH, CH_3_OH and CH_4_ can be formed from CO_2_ reduction on a Co-based porphyrin. Figure 1a,d,g measured at pH=1 shows that the reduction current is accompanied by the simultaneous formation of H_2_ and CH_4_. The *m/z*=28 signal in Fig. 1 was not corrected for the CO_2_ fragmentation, and therefore the CO signal combines CO production from CO_2_ electroreduction with CO formation from CO_2_ fragmentation in the mass spectrometer (MS). This explains why the CO signal decreases for more negative potentials at which the CO_2_ reduction rate is higher, as a result of the lower local CO_2_ concentration near the electrode surface. However, at pH=2 and 3, an increase in the CO signal with more negative potential is observed, simultaneously with the CH_4_ production, suggesting that CO is an intermediate in the reaction (as also suggested by the fact that CO may be reduced to CH_4_ on CoPP-PG; Fig. 4 below). Most significantly, at pH=3, CO and CH_4_ production is observed at less-negative potentials than H_2_ evolution, showing that the CO_2_ reduction has a different pH dependence from the hydrogen evolution reaction. We chose to restrict ourselves to pH≤3 in perchlorate solution in order to avoid the interference of buffering anions such as bicarbonate or phosphate (see below) with the CO_2_ reduction process.

We have performed a number of experiments to convince ourselves that the Co-PP is indeed the active catalytic centre turning over dissolved CO_2_. On the unmodified PG electrode and on a PG electrode modified with Co-free protoporphyrin, H_2_ evolution was observed, but no CO_2_ reduction (Supplementary Figs 5 and 6). A PG electrode onto which a small amount of Co was electrodeposited was also tested for CO_2_ reduction, but showed no activity (Supplementary Fig. 7). Finally, the reduction of isotopically labelled ^13^CO_2_ in deuterated water yielded *m/z*=19 (corresponding to ^13^CD_3_) as reduction product (Supplementary Fig. 8), which irrefutably proves the reduction of dissolved CO_2_ into methane. These combined results show that the immobilized Co protoporphyrin is responsible for the production of CO and methane from CO_2_ electroreduction.

**Figure 2:**
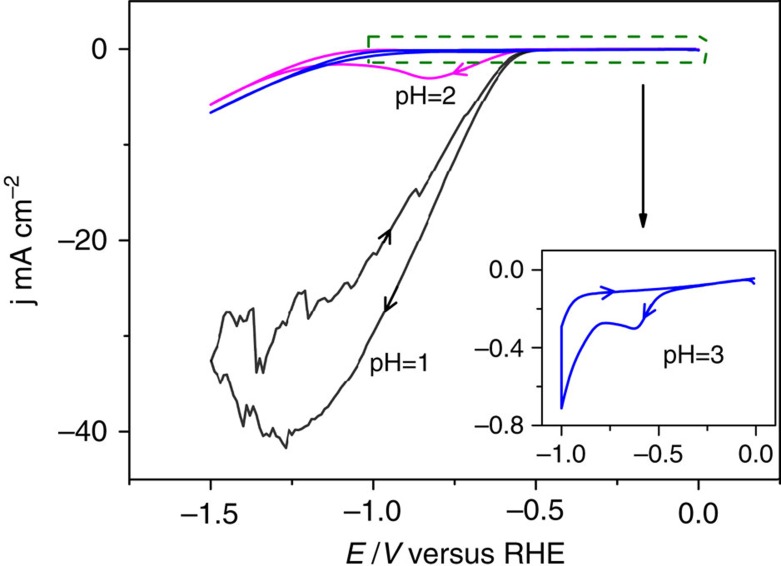
pH dependence of hydrogen evolution reaction on the CoPP-PG electrode. Hydrogen evolution reaction at pH=1 (black curve), pH=2 (red curve) and pH=3 (blue curve) on Co protoporphyrin-modified PG electrode in the absence of CO_2_. Inserted: highlight of the voltammetry at pH=3. Scan rate was in all cases is 100 mV s^−1^. All electrolyte solutions were 0.1 M perchlorate, with different ratios of H^+^ and Na^+^.

**Figure 3:**
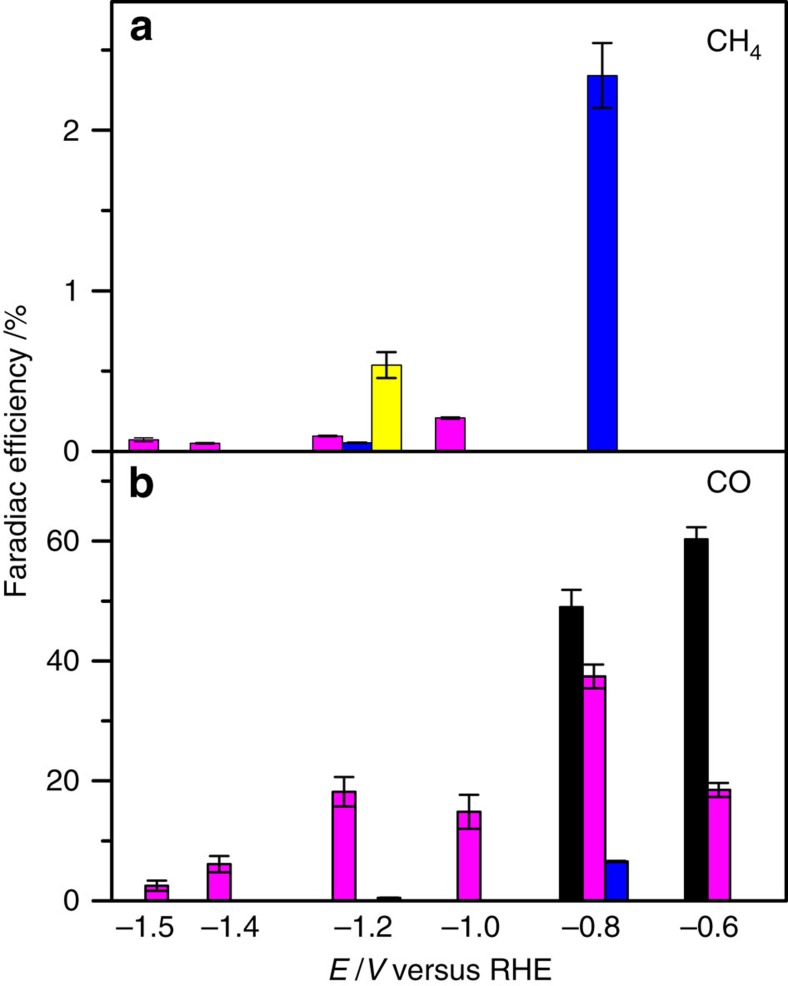
FE of carbon dioxide reduction to CO and methane. FEs to CO and CH_4_ were determined for yellow bars: pH=1, *P*_CO2_=1 atm; blue bars: pH=1, *P*_CO2_=10 atm; magenta bars: pH=3, *P*_CO2_=1 atm and black bars pH=3, *P*_CO2_=10 atm. FE of (**a**) CH_4_ and (**b**) CO in 0.1 M perchlorate solution saturated with CO_2_. At each potential, the electrolysis was conducted for 1 h at *P*_CO2_=1 atm, while it is 90 min at *P*_CO2_=10 atm due to the longer time to reach the steady state. Error bars were determined from 3–8 data points based on samples taken every 6 min during the steady state of a single electrolysis run.

As mentioned, the most important conclusion from Fig. 1 is the remarkable role of the pH. Initially, we performed the CO_2_ reduction experiments at pH=2 and 3 in buffered phosphate solution, also yielding methane as a product but with a pH dependence that was not straightforward to understand. Therefore, we decided to remove the buffering phosphate anions, as they are suspected to interfere with the reactivity by coordinating to the catalytic centre^24^ or interacting with the catalytic intermediates. In non-adsorbing perchlorate solution, the role of the proton concentration can be better understood by comparing the voltammetry of the CoPP-PG in the absence of CO_2_ at pH=1–3, as shown in Fig. 2. At pH=1, there is only a single catalytic reduction wave in the potential window studied, corresponding to the reduction of H^+^ to H_2_. The voltammetry at pH=2 and 3 shows two waves, one at less-negative potential that is proportional to the H^+^ concentration and corresponds to H^+^ reduction, and one starting at −1.1 V that corresponds to H_2_O reduction. This is also reflected in the H_2_ formation profiles observed in the mass signals in Fig. 1. We must also take into account here that because of the relatively low proton concentration at pH=3, the direct proton reduction quickly runs into diffusion limitations, and further H_2_ evolution can only take place at more negative potentials by direct water reduction, which does not suffer from such diffusion limitations. By comparing the results in Figs 1 and 2, we conclude that H_2_ evolution dominates over CO_2_ reduction in the presence of a high concentration of protons in solution, whereas the opposite is the case for pH=3. The activation of CO_2_ is apparently less sensitive to the presence of protons, implying that water molecules are just as powerful in hydrogenating the activated CO_2_. This remarkable pH dependence is somewhat similar to observations made by Noda *et al*.^25^ during CO_2_ reduction on a gold electrode. The important new finding here is that this small pH shift is the key in favouring CO_2_ reduction over H_2_ evolution, also on our molecular catalyst, especially in the absence of buffering anions. This is also evidenced by the FE measurements summarized in Fig. 3, to be discussed next. A mechanistic explanation for this pH sensitivity will be given in the Discussion section.

### Faradaic efficiency

The FE for the simultaneous CO_2_ and water reduction to hydrogen, CO and methane was determined separately with long-term electrolysis experiments, using a gas chromatography setup coupled to an electrochemical cell, as detailed elsewhere^26^,^27^. Figure 3 shows results for CO and CH_4_ at pH=1 and 3 for different potentials. The remaining current is used to form H_2_. The quantitative data and error bars are summarized and further explained in Supplementary Table 1. HCOOH was also observed as a minority product at pH=1 using HPLC, but was not observed at pH=3 (Supplementary Fig. 3). As mentioned above, methanol was observed as a product using OLEMS (Supplementary Fig. 2), but it remained below the detection limit during the gas chromatography (GC) measurements. At pH=1, the FE to CO and methane is low, on the order of a per cent, and the dominant product is H_2_, and therefore for pH=1, we show results at only a single potential in Fig. 3. Note, however, that at pH=1, more methane is produced than CO. At pH=3, a dramatic change in selectivity is observed, with now CO being a majority product, especially at less cathodic potentials, for which the FE to CO is ∼40%. This high selectivity is maintained for at least 1 h during the long-term electrolysis experiment at fixed potential (Supplementary Fig. 9), testifying to the good stability of the catalyst. The stability and integrity of the CoPP-PG electrode was also confirmed by pre- and post-electrolysis analysis using X-ray Photoelectron Spectroscopy (XPS), Raman and nuclear magnetic resonance (Supplementary Figs 10–12). Raman spectroscopy showed no significant change in the spectral features of the CoPP-PG surface; XPS showed no change in Co oxidation state after 1 h of electrolysis; and nuclear magnetic resonance showed no decomposition products in solution that could be related to CoPP. Figure 3 also illustrates that less methane is produced at pH=3 as compared with pH=1. We ascribe this lower methane production to the slower reduction of CO to CH_4_ at pH=3 compared with pH=1 (see next paragraph). The efficiency towards CO can be further boosted by performing the experiment at higher CO_2_ pressure. Figure 3 illustrates this for a CO_2_ pressure of 10 atm, which leads to a FE of ∼ 60% at pH=3 at a potential of −0.6 V. Note that at pH=1, both the efficiency towards CO and CH_4_ increases to a few % when the reduction is carried out at increased CO_2_ pressure. We emphasize that OLEMS and GC experiments exhibited good consistency and reproducibility. The error bars shown in Fig. 3 were based on single long-term electrolysis experiments sampled every 6 min.

### Reduction of other compounds

**Figure 4:**
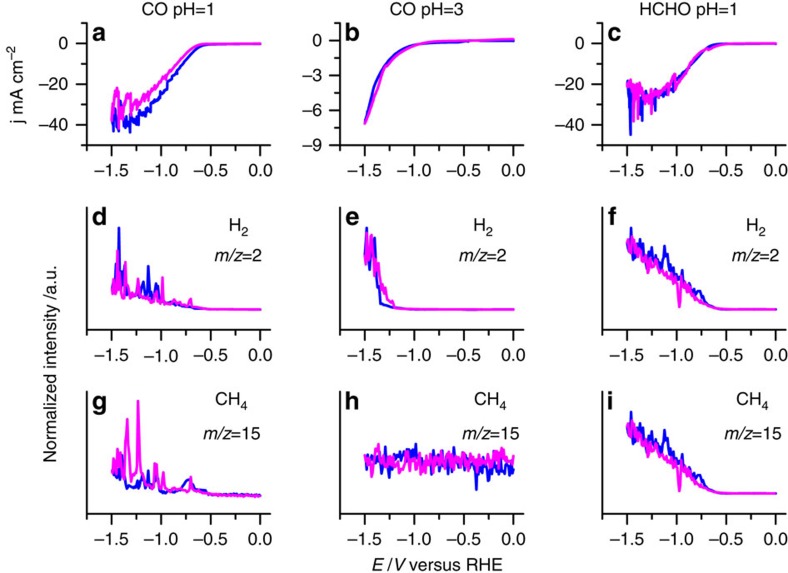
Identification of volatile products by OLEMS during electrochemical reduction of CO and HCHO. CV of CO reduction in (**a**) 100 mM HClO_4_ and (**b**) 1 mM HClO_4_+99 mM NaClO_4_ saturated with CO with associated mass fragments of volatile products detected with OLEMS. (**c**) CV of HCHO (5 mM) reduction in 100 mM HClO_4_ with associated mass fragments measured with OLEMS. (**d**–**f**) The corresponding OLEMS signals for *m/z*=2 (H_2_); (**g**–**i**) The corresponding OLEMS signals for *m/z*=15 (CH_4_). Scan rate: 1 mV s^−1^. Blue lines are negative-going (forward) scans; magenta lines are positive-going (return) scans. Supplementary Fig. 14 shows the same data with the unnormalized MS signals, as well as the signals obtained in the first and second CV scan.

To determine the involvement of potential intermediates, we also studied the reduction of HCOOH, CO and formaldehyde (HCHO), by combined voltammetry-OLEMS. HCOOH was not reduced at either pH=1 or 3 (Supplementary Fig. 13), and is therefore an end product, not an intermediate. Figure 4 shows the voltammetry and associated OLEMS mass signals on the CoPP-PG electrode for CO reduction at pH=1 and 3, and for HCHO reduction at pH=1. Remarkably, CO is clearly reduced to methane at pH=1, simultaneous with H_2_ evolution, but the CO reduction activity is much lower compared with hydrogen evolution at pH=3, with an insignificant amount of CH_4_ detected. This observation is consistent with the results in Fig. 3, showing that methane production from CO_2_ is lower at pH=3. HCHO is reduced to methane at pH=1 and 3 (Fig. 4 only shows pH=1). Interestingly, HCHO is not reduced to significant amounts of methanol, whereas methanol is the product of HCHO reduction on copper electrodes^6^. Figure 4 suggests that CO and HCHO, or their catalyst-bound derivatives, are intermediates in the reaction mechanism from CO_2_ to CH_4_, but HCOOH is not. It also shows that the reduction of CO exhibits a different pH dependence compared with CO_2_ reduction, explaining why the selectivity of CO_2_ towards CO increases with higher pH, but the selectivity towards CH_4_ decreases with higher pH.

## Discussion

**Figure 5:**
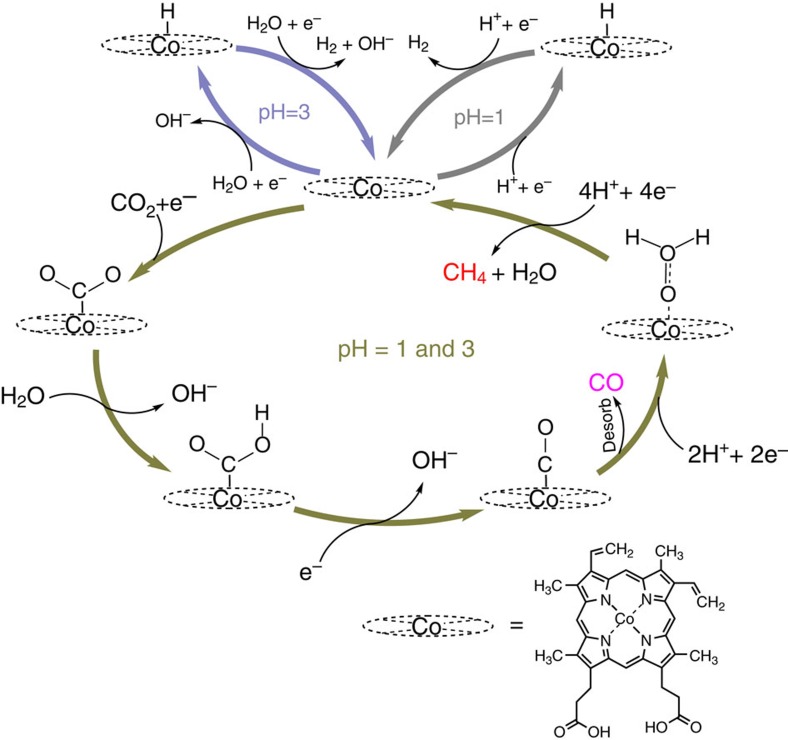
Proposed mechanistic scheme for the electrochemical reduction of CO_2_ on Co protoporphyrin. H^+^ and H_2_O are the hydrogen source for the hydrogen evolution reaction at pH=1 and 3, respectively. CO_2_^·−^ is the initial intermediate for the CO_2_ reduction to CO. CO can be further reduced to methane with HCHO as an intermediate. The catalytically inactive ‘resting' state of the Co is assumed to be 2+. The reduction of Co^2+^ to Co^+^ is supposed to trigger both the H_2_ evolution and CO_2_ reduction pathways.

The results presented above give unique new insights into the mechanism of CO_2_ electroreduction on immobilized Co protoporphyrins, and the observed pH dependence reveals the important role of the initial electron transfer to CO_2_ in the overall mechanism as explained below, and as illustrated in our suggested mechanistic scheme in Fig. 5. At pH=1, the dominant reaction is hydrogen evolution:


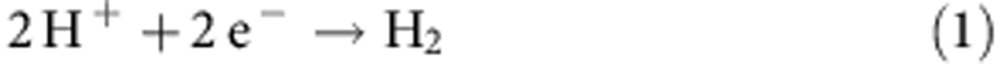


At pH=3, the main origin of hydrogen evolution is direct water reduction:


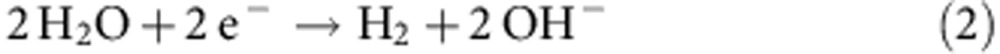


with reaction 1 generating a smaller amount of H_2_ at less-negative potential due to diffusion limitations (Fig. 2). This observation is very similar to recent experiments on platinum electrodes^28^. The observation that CO_2_ reduction to CO becomes much more dominant at higher pH, must mean that CO_2_ activation does not sensitively depend on the presence of protons, and hence must involve an intermediate that can easily react with water at any pH. Such an intermediate is most likely a negatively charged Brønsted base, and the most obvious candidate for this intermediate is a CO_2_ radical anion^25^,^29^,^30^ bound to the Co complex ‘M':


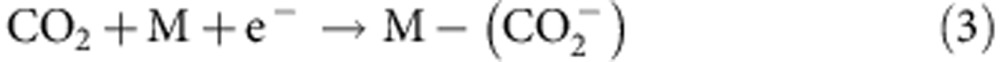


which subsequently reacts with water to a metal-bound carboxyhydroxyl intermediate:


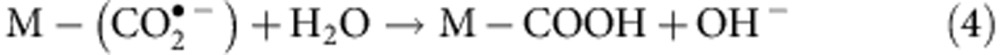


The formation of the CO_2_^·−^ radical anion normally has a very negative redox potential^3^,^8^, but may be shifted to less-negative potential by the stabilization provided by the coordination of CO_2_^·−^ to the catalyst. The carboxyhydroxyl intermediate then generates CO:


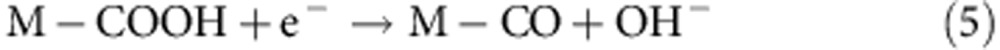


with the CO subsequently dissociating from the complex. Owing to the presence of the negatively charged intermediate in reaction 4, the pH dependence of this pathway is different from that of the mechanism for reactions 1 and 2, in which no such intermediate is assumed. For reactions 1 and 2, we assume:


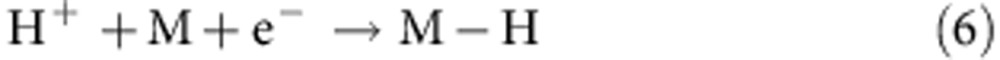



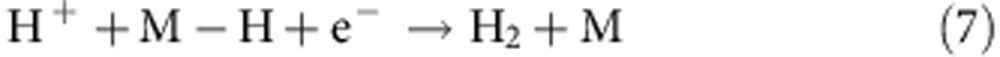


and


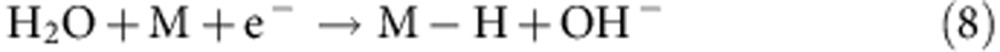



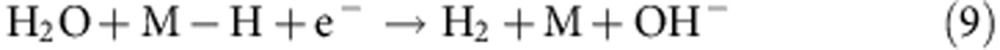


which involve concerted proton-coupled electron transfer at every step^31^,^32^. Reaction 4 is different from the reaction suggested by the Density Functional Theory (DFT) calculations of Leung *et al*.^29^,^30^ because we specify that the proton donor may be water, rather than H^+^, owing to the basic character of the CO_2_ radical anion intermediate. Note that in this mechanism, the reaction rate for CO_2_ reduction itself does not depend on pH, only its relative rate with respect to the hydrogen evolution. Another way of formulating our mechanism is by stating that in the potential window of interest, CO_2_ reduction is approximately zeroth order in proton concentration, while hydrogen evolution is first order in proton concentration.

The further reduction of CO must be slower than its generation, explaining the relatively low overall FE of CO_2_ reduction to methane. To explain the pH dependence of CO reduction and methane selectivity from CO_2_, we must assume that CO is reduced to methane without the involvement of negatively charged intermediates. Our experiments also show that an intermediate or by-product of CO reduction to methane is HCHO. Our suggested overall mechanism is summarized in Fig. 5.

The above mechanism, which we believe explains our observations consistently, has important implications for future catalyst design. The onset potential for CO_2_ reduction is determined by reaction 3, that is, by the stabilization of the CO_2_ radical anion coordinated to the complex. As noted above, the onset potential appears to be related to the Co^2+^/Co^+^ redox transition on the basis of CV^19^ and also on the previous observation that the Co^+^ state is the active state for proton reduction^22^. Nielsen and Leung have also concluded, based on literature data and their own DFT calculations, that CO_2_ binds to the Co^+^ state of the porphyrin^29^,^30^. Therefore, we assume that Co^+^ state of the CoPP is the catalytically active state. The closer the Co^2+^/Co^+^ redox potential lies to the overall equilibrium potential, the lower is the overpotential for CO_2_ reduction. Reaction 3 is therefore the potential-determining step^33^,^34^. The key point is that the formation of this intermediate is decoupled from proton transfer, as otherwise we cannot explain the observed pH dependence, an important feature not included in the recent DFT calculations of Tripkovic *et al*.^9^. Therefore, future calculations must take into account the existence of such intermediates, and should aim at enhancing the stability of the intermediate in reaction 3. Moreover, in order to have a higher overall efficiency towards methane, the rate of the reduction of CO to methane must be enhanced. Presumably, the rate of this reaction can be tuned by the binding of CO to the complex. This will also require further experiments and calculations aimed at screening various catalyst alternatives. We also believe that our mechanism provides a possible rationale for tuning the H_2_/CO ratio from electrochemical CO_2_ reduction, as was recently reported for a Ru-based molecular catalyst in aqueous solution^35^.

A final word on the overpotential and the TOFs of our catalyst in comparison with previous work on molecular catalysts for CO_2_ electroreduction to CO. From our experiment, we calculate TOFs through the formula: (FE for CO production) × (current density/2*F*)/(number of Co-PP per cm^2^), where *F*=Faraday constant. In Fig. 3, the average current densities measured over 1 h at potentials of −0.6 and −0.8 V versus RHE, corresponding to overpotentials of ca. 0.5 and 0.7 V, were 0.08 and 0.16 mA cm^−2^ (at atmospheric pressure), respectively. This corresponds to TOFs of ca. 0.2 and 0.8 s^−1^. Costentin *et al*.^10^ have recently reported on the enhanced activity of a modified Fe tetraphenylporphyrin for CO_2_ reduction to CO in a mixed DMF–water solvent. In their experiment, the porphyrin was in solution. Their measured current densities and corresponding effective CO_2_ turnover rates are very similar to ours, namely, 0.3 mA cm^−2^ (see Supplementary Fig. 5 in their paper) at a similar overpotential of ca. 0.5 V. Note that this comparison does not take into account that the solubility of CO_2_ is considerably higher in DMF–water mixtures than in water^36^, thereby leading to correspondingly higher turnover rates in the DMF–water mixture. From a mathematical model for their reactive system including mass transport of the catalyst to the electrode surface, they report a catalytic TOF of ca. 3,000 s^−1^. This is a TOF of a homogeneous catalyst corrected for the slow mass transport in their system, and can therefore not be compared directly with the ‘effective' TOF of our heterogeneous catalyst. However, from the similar real current densities at a similar overpotential, we believe that we can safely state that our immobilized catalyst system has a similar efficiency.

Summarizing, we have shown that a Co protoporphyrin immobilized on a PG electrode can reduce CO_2_ to CO and even to the 6- and 8-electron products methanol and methane, in a purely aqueous electrolyte phase, with a moderate overpotential of ca. 0.5 V. The efficiency of our catalyst (that is, effective rate at given overpotential) compares favourably with best porphyrin-based catalyst reported in the literature^10^. For optimal FE, that is, low concomitant H_2_ production, the proton concentration needs to be suitably tuned to the CO_2_ concentration. The pH-dependent activity and selectivity are explained by a mechanism in which the initial step of CO_2_ reduction leads to a catalyst-bound CO_2_^·−^ radical anion. This intermediate has a strong Brønsted-base character and can abstract a proton from water, thereby leading to an overall reactivity of the CO_2_ reduction whose pH dependence is substantially different from the competing H_2_ evolution. Lowering the potential for the formation of this catalyst-bound CO_2_^·−^ radical anion is therefore the key to making a better catalyst with a lower overpotential, and a suitable adjustment of pH will contribute significantly to a high FE of such a catalyst. The further reduction of CO to methane and methanol is slow owing to the weak binding of CO to the catalyst, and owing to the fact that CO reduction prefers a more acidic environment. These new insights into the mechanism of CO_2_ reduction on immobilized molecular catalysts in aqueous solution provide important design rules for future catalyst improvement.

## Methods

### Electrochemistry and chemicals

The experiments were performed on home-made PG electrodes (Carbone-Lorraine; diameter, 5 mm). Before each experiment, the electrodes were polished using P500 and P1000 SiC sandpaper consecutively, and were ultrasonicated in ultrapure water (Milli-Q gradient A10 system, 18.2 MΩ cm) for 1 min and dried in a flow originating from compressed air. The electrodes were subsequently immersed in the Co protoporphyrin (Frontier Scientific) solution (0.5 mM in borate buffer) for 5 min to immobilize the protoporphyrin on the surface and rinsed with ultrapure water before the experiments. A one-compartment electrochemical cell was used, with a platinum flag as counter electrode and a RHE as a reference, to which all potentials in this work are referred. The reference electrode was separated from the working electrode compartment through a Luggin capillary. An Ivium potentiostat/galvanostat (IviumStat) was used for the electrochemical measurements. Solutions were prepared from HClO_4_ (Merck, 70%), NaClO_4_ (Sigma-Aldrich, ≥98.0%), NaOH (Sigma-Aldrich, 99.998%), borate (Sigma-Aldrich) and ultrapure water. Argon (Hoekloos, purity grade 6.0) was purged though the solutions for 30 min before the experiment to remove dissolved oxygen. The reported current densities refer to the geometric surface area.

### Online electrochemical mass spectrometry

The volatile products of the CO_2_ electrochemical reduction were detected using online electrochemical mass spectroscopy (OLEMS) with an evolution mass spectrometer system (European Spectrometry systems Ltd)^23^. A porous Teflon tip (inner diameter, 0.5 mm) with a pore size of 10–14 μm was positioned close (∼10 μm) to the centre of the electrode. Before the experiments, the tip was dipped into a 0.2-M K_2_Cr_2_O_7_ in 2 M H_2_SO_4_ solution for 15 min and rinsed with ultrapure water thoroughly. The gas products were collected through a polyether ether ketone (PEEK) capillary into the mass spectrometer. A 2,400-V secondary electron multiplier (SEM) voltage was applied for all the fragments except for hydrogen (*m/z*=2) which is 1,500 V. The OLEMS measurement was conducted while CV was scanning from 0 to −1.5 V and back at a scan rate of 1 mV s^−1^.

### Gas chromatography

The quantitative measurements of the gas products were carried out using GC^26^,^27^. At atmospheric pressure, CO_2_ was continuously purged through a two-compartment flow cell with a volume of 12 ml for each compartment at a rate of 5 ml min^−1^ for 30 min to saturate the electrolyte. The flow rate declined to 2 ml min^−1^ while a constant potential was applied for 1 h. The reference electrode used here is a Ag/AgCl electrode. The experiments at high CO_2_ pressure (*P*=10 atm) were conducted in a stainless-steel autoclave using a Pt mesh as a counter electrode, and a home-made Ag/AgCl in 3 M KCl as a reference electrode. All potentials were scaled to RHE after the experiments for both atmospheric and high pressure, with *E*(versus Ag/AgCl)=*E*(versus RHE)−0.197 V−pH × 0.059. CO_2_ was continuously purged through the autoclave before and during the electrolysis with a flow rate of 50 ml min^−1^. The reactor effluent was sampled via GC once every 6 min. CO, CO_2_, H_2_ and hydrocarbons were simultaneously separated using two series columns in series (a ShinCarbon 2 m micropacked column and a Rtx-1 column). The quantitative analysis of the gas products was performed using a thermal conductivity detector (H_2_ and CO) and flame ionization detector (hydrocarbons).

### Online HPLC

HPLC (Prominence HPLC, Shimadzu) was used to detect liquid products produced during electrochemical reduction of CO_2_ using a method described in previous work^37^. Samples were collected using a Teflon tip (inner diameter: 0.38 mm) positioned ∼10 μm from the centre of the electrode surface (diameter: 1 cm). The sample volume collected was 60 μl stored in a 96-well microtitre plate (270 μl per well, Screening Device b.v.) using an automatic fraction collector (FRC-10A, Shimadzu). The flow rate of the sample collection was adjusted to 60 μl min^−1^ with a Shimadzu pump (LC-20AT). A linear sweep voltammogram was recorded while the sample was collecting at a scan rate of 1 mV s^−1^ from 0 to −1.5 V versus RHE. The microtitre plate with collected samples was then placed in an auto-sampler (SIL-20A) holder and 30 μl of sample was injected into an Aminex HPX 87-H (Bio-Rad) column. The eluent was diluted sulfuric acid (5 mM) with a flow rate of 0.6 ml min^−1^. The temperature of column was maintained at 85 °C using a column oven (CTO-20A) and the separated compounds were detected with a refractive index detector (RID-10A).

## Additional information

**How to cite this article:** Shen, J. *et al*. Electrocatalytic reduction of carbon dioxide to carbon monoxide and methane at an immobilized cobalt protoporphyrin. *Nat. Commun.* 6:8177 doi: 10.1038/ncomms9177 (2015).

## Supplementary Material

Supplementary Information Supplementary Figures 1-14, Supplementary Table 1 and Supplementary References
