# Supplementary material for: Electrocatalytic reduction of carbon dioxide to carbon monoxide and methane at an immobilized cobalt protoporphyrin
Source: Nat Commun. 2015 Sep 1;6:8177. doi: 10.1038/ncomms9177 (PMC4569799; doi:10.1038/ncomms9177)
Supplement: Supplementary Information — Supplementary Figures 1-14, Supplementary Table 1 and Supplementary References [file ncomms9177-s1.pdf]

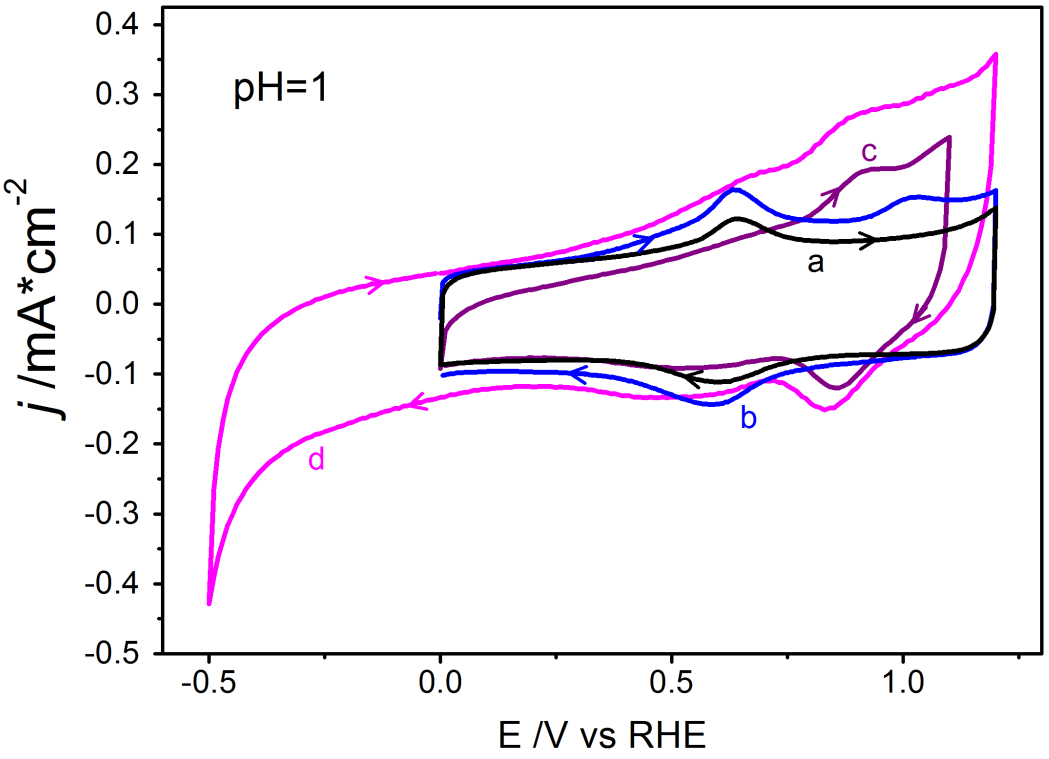

Supplementary Figure 1. Characterization of immobilized cobalt protoporphyrin electrode. The cyclic voltammogram of: (a) pyrolytic graphite electrode; (b) pyrolytic graphite electrode with 100 μL 0.5 mM cobalt protoporphyrin solution in electrolyte; (c) cobalt protoporphyrin immobilized pyrolytic graphite electrode and (d) cobalt protoporphyrin immobilized pyrolytic graphite electrode scanned to more negative potential. All voltammograms obtained in 0.1 M $HClO_4$ solution; scan rate is 500 mVs^{-1}. From the peak corresponding to the Co^{3+}/Co^{2+} redox couple at 0.8-0.9 V (with due account of the capacitive background current), the coverage of cobalt protoporphyrin can be estimated as $4x10^{-10}$ mol cm^{-2}. The cleanliness of the solution was verified by blank voltammetry of a platinum electrode, which showed no impurities.

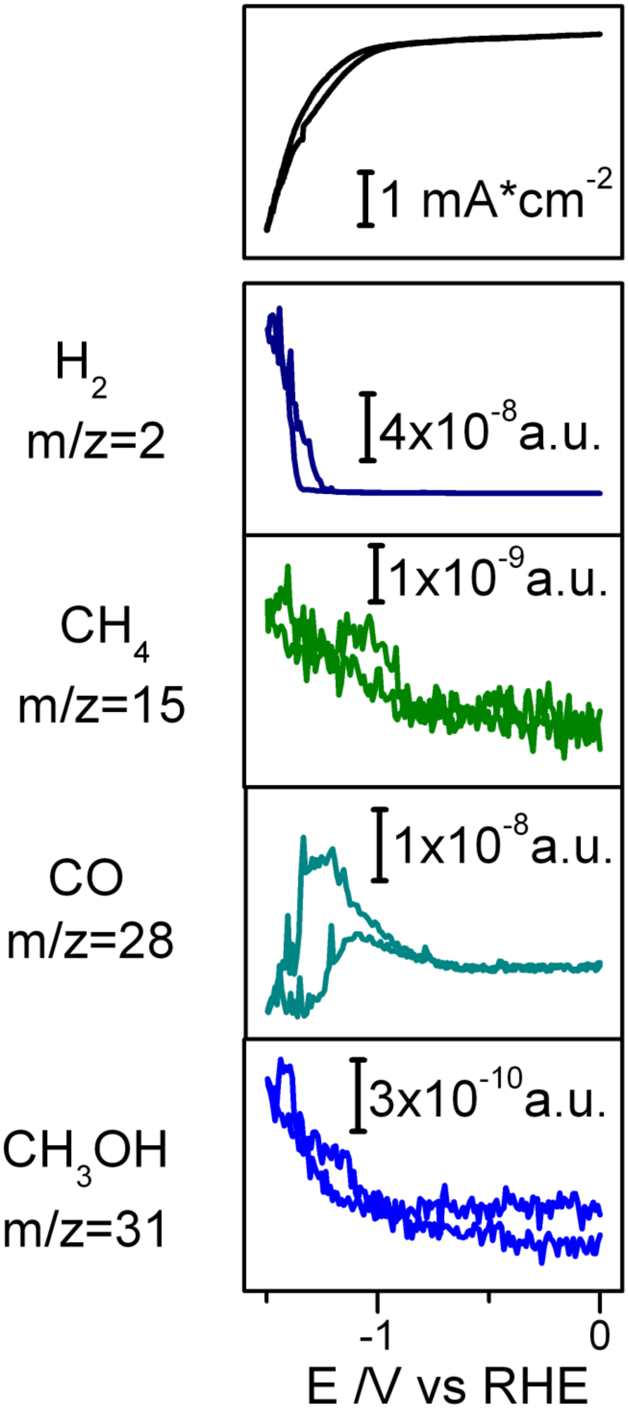

Supplementary Figure 2. Cyclic voltammetry combined with OLEMS measurement of electrochemical reduction of CO_2 on a cobalt-tetramethoxyphenylporphyrin modified pyrolytic graphite electrode in 1 mM $HClO_4$ + 99 mM $NaClO_4$ (pH=3) solution, showing the cyclic voltammetry (upper panel) and the hydrogen, methane and methanol formation (lower panels). This experiment demonstrates that a small amount of alcohol (m/z=31) is produced from the CO_2 reduction catalyzed by the cobalt porphyrin. From the absence of higher alcohols C_2 signals, we conclude that m/z=31 must correspond to methanol.

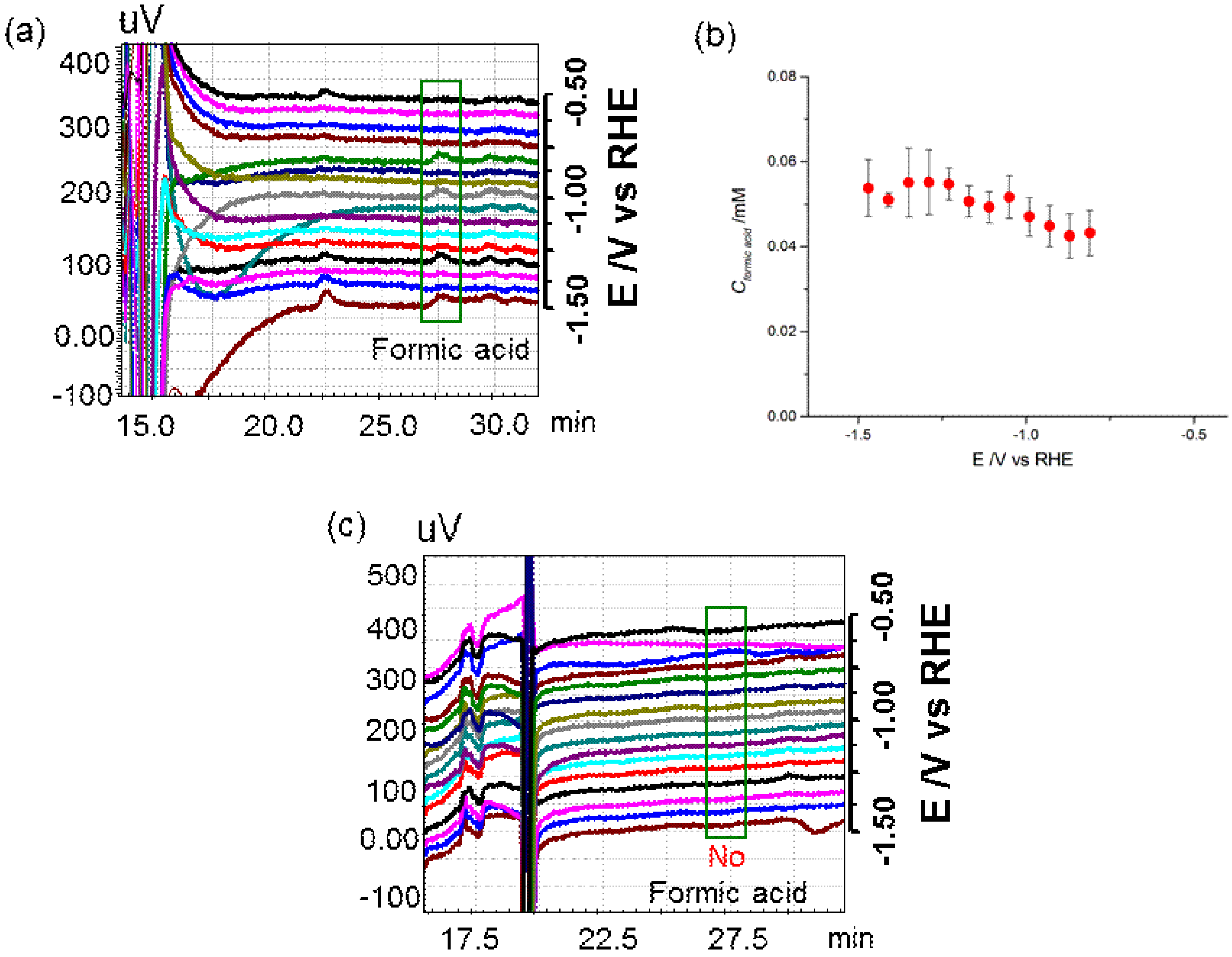

Supplementary Figure 3. Liquid products detected using HPLC. (a) Chromatograms obtained during the electrochemical reduction of CO_2 on the CoPP-PG electrode in 0.1 M $HClO_4$ solution vs potential, which were collected every 60 mV. (b) The corresponding concentration of formic acid vs potential at pH=1; error bars determined from 3 separate experiments. (c) Chromatograms for electrochemical reduction of CO_2 on the CoPP-PG electrode in 1 mM $HClO_4$+ 99 mM $NaClO_4$ solution vs potential, which were collected every 60 mV, showing the absence of formation of formic acid at pH=3. The fluctuations of the detected HCOOH in Fig.(b) are due to the very small amount of formic acid formed from CO_2 electrochemical reduction in combination with the hydrogen formation and sample collection.

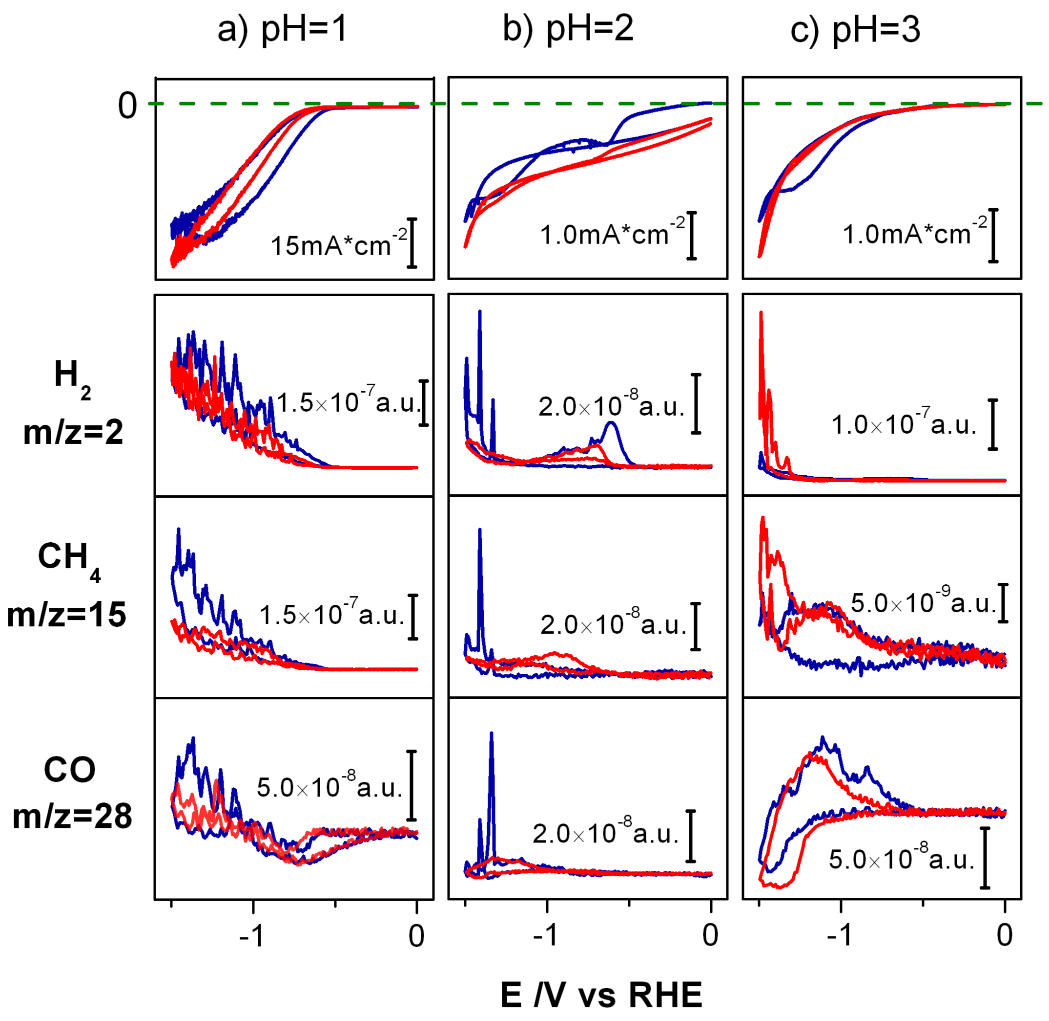

Supplementary Figure 4. Voltammetry and the unnormalized signals of the volatile product identification by OLEMS during the electrochemical reduction of CO_2 on cobalt protoporphyrin immobilized pyrolytic graphite electrode. (a) 0.1 M $HClO_4$ (b) 10 mM $HClO_4$ + 90 mM $NaClO_4$ and (c) 1 mM $HClO_4$ + 99mM $NaClO_4$; showing first cycle (blue curve) and second cycle (red curve). Top panels: cyclic voltammetry. Lower panels: associated OLEMS mass signals for m/z=2 (H_2), 15 (CH_4) and 28 (CO). Scan rate in all cases 1 mV s^{-1}.

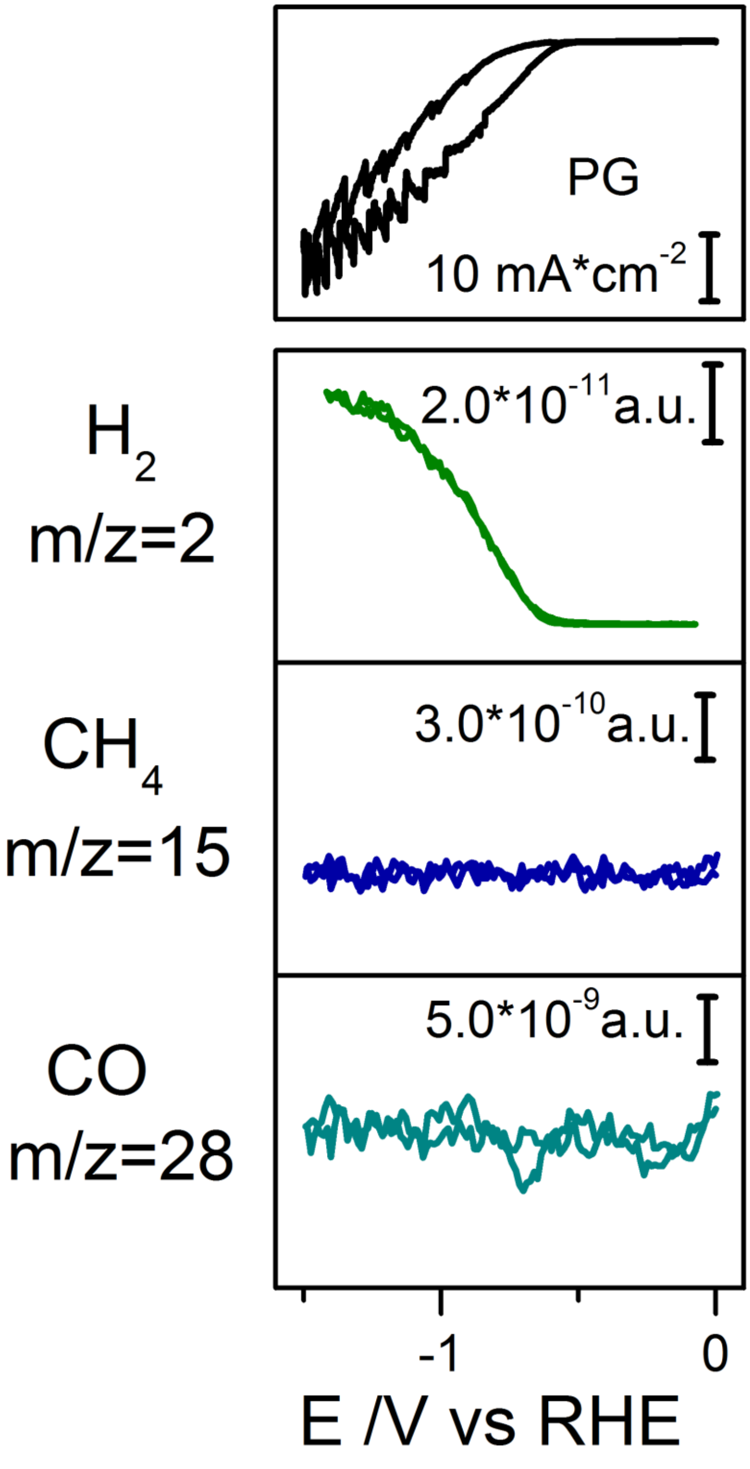

Supplementary Figure 5. Combined CV-OLEMS measurement of electrochemical reduction of CO_2 on an unmodified pyrolytic graphite electrode in 0.1 M $HClO_4$ solution, showing the cyclic voltammetry (upper panel) and the hydrogen, methane, and carbon monoxide formation (lower panels). This experiment demonstrates that the catalytic activity towards CO_2 reduction is not from pyrolytic graphite.

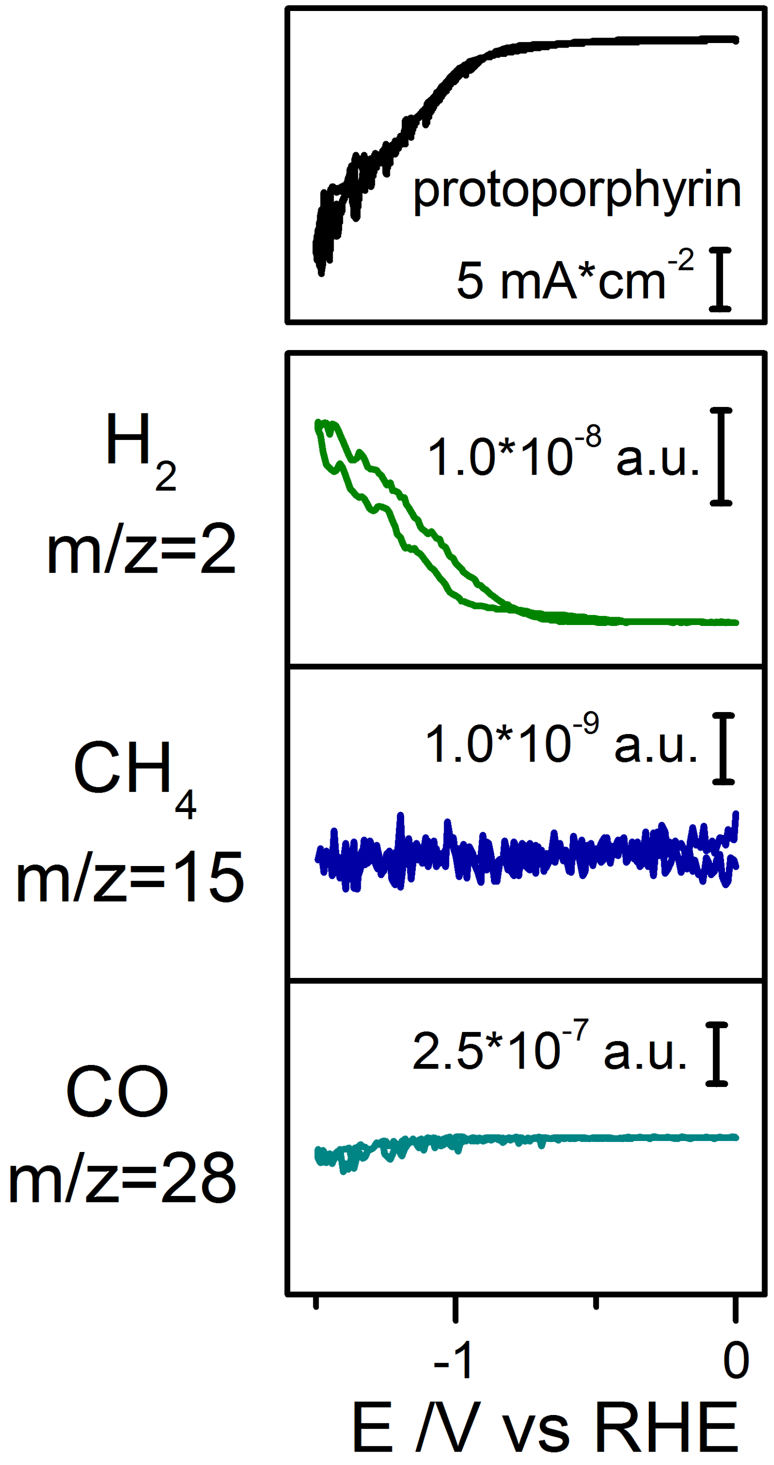

Supplementary Figure 6. Combined CV-OLEMS measurement of electrochemical reduction of CO_2 on cobalt-free protoporphyrin modified pyrolytic graphite electrode in 0.1 M $HClO_4$ solution, showing the cyclic voltammetry (upper panel) and the hydrogen, methane and carbon monoxide formation (lower panels). This experiment demonstrates that the catalytic activity for CO_2 reduction comes from the interaction of the cobalt metal center and protoporphyrin ring.

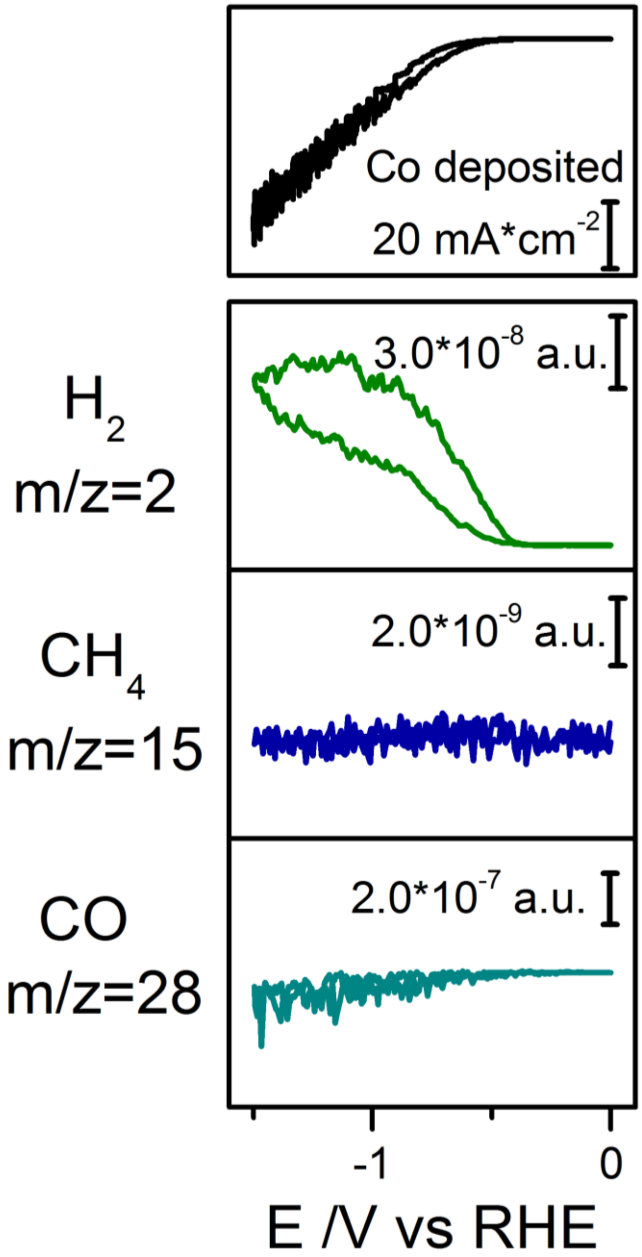

Supplementary Figure 7. Combined CV-OLEMS measurement of electrochemical reduction of CO_2 on a cobalt electrodeposited pyrolytic graphite electrode in 0.1 M $HClO_4$ solution, showing the cyclic voltammetry (upper panel) and the hydrogen, methane and carbon monoxide formation (lower panels). This experiment demonstrates that the catalytic activity for CO_2 reduction is not from the cobalt released from cobalt protoporphyrin. The deposition of cobalt was conducted using the same procedure as described elsewhere[1]. The solution used for cobalt electrodeposition was 3.5 mM $CoCl_2$ in 1 M NH_4Cl solution (pH=4.5). Electrochemical experiments were conducted in a one-compartment three-electrode electrochemical cell with a pyrolytic graphite electrode (diameter:5mm) as working electrode, a graphite rod as a counting electrode and a KCl saturated Ag/AgCl electrode as a reference electrode, to which all potentials here were referred. Cyclic voltammetry was conducted from 0.4 V to -1.3 V to verify the electrochemistry of the working electrode in the electrodeposition bath, after which a current-time transient experiment was conducted by starting the potential at 400 mV for 60s, and jumping to first potential step at -1.3 V for 2s with an interval of 0.2s.

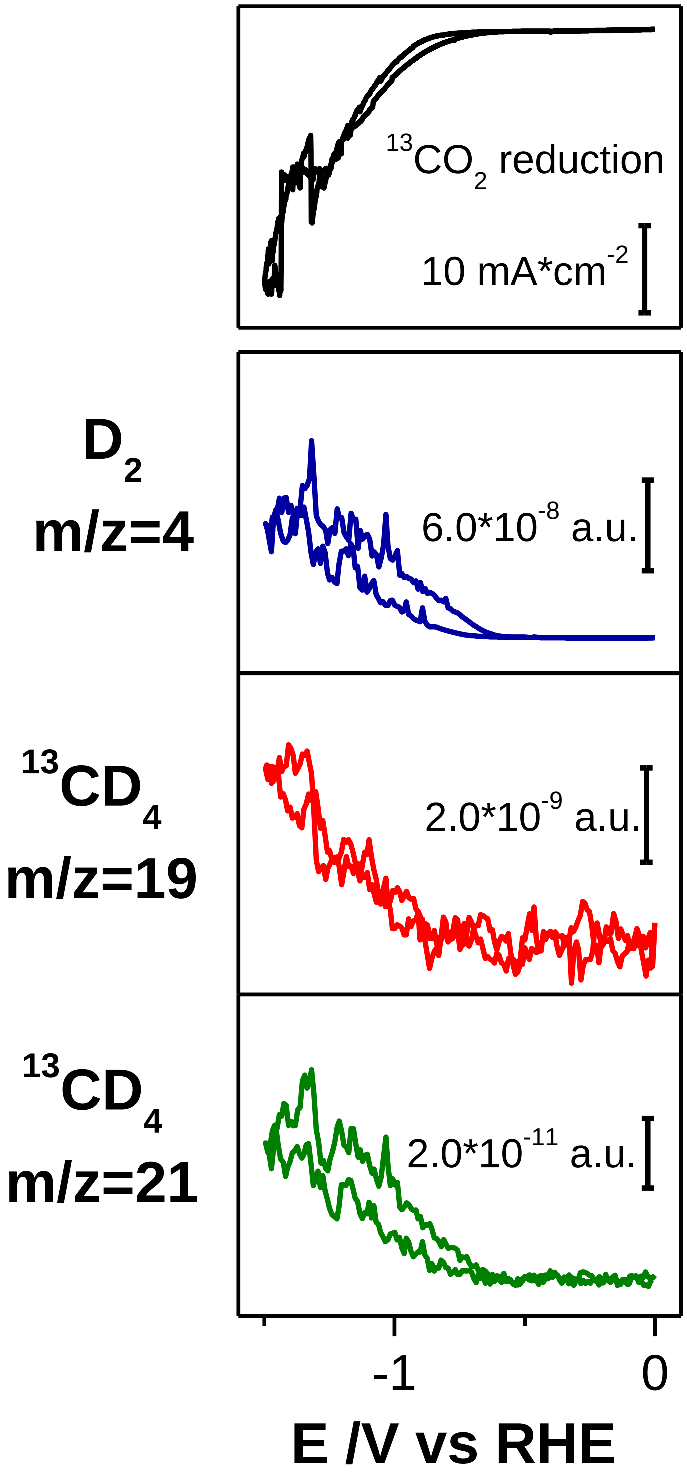

Supplementary Figure 8. Combined CV-OLEMS measurement of electrochemical reduction of $^{13}CO_2$ on cobalt protoporphyrin modified pyrolytic graphite electrode in 0.1 M $DClO_4$ + D_2O solution, showing the cyclic voltammetry (upper panel) and the hydrogen and methane formation (lower panels). This experiment proves that the catalytic activity of cobalt protoporphyrin and the formation of methane are from dissolved CO_2. m/z=19 corresponds to $^{13}CD_3$; m/z=21 corresponds to $^{13}CD_4$.

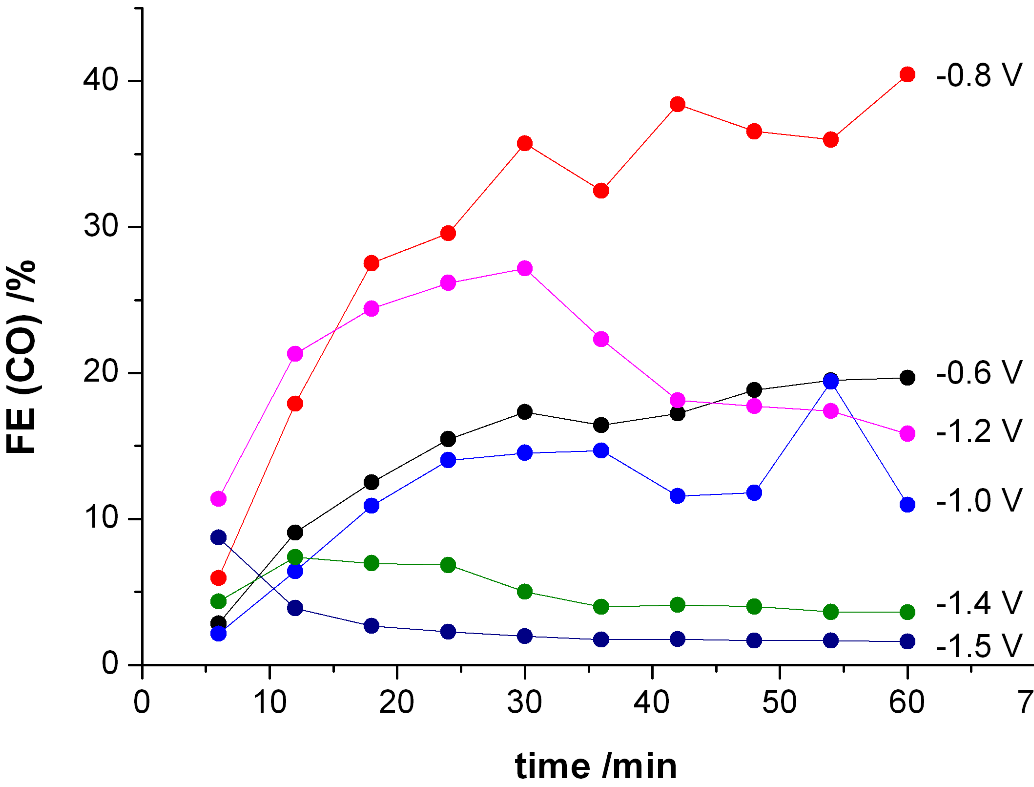

Supplementary Figure 9. Faradaic efficiency (FE) of CO from controlled potential electrolysis of CO_2 electrochemical reduction on cobalt protoporphyrin modified pyrolytic graphite electrode at different potentials vs RHE as a function of time. Solution: 1 mM $HClO_4$ + 99 mM $NaClO_4$ (pH=3); CO_2 pressure: 1 atm.

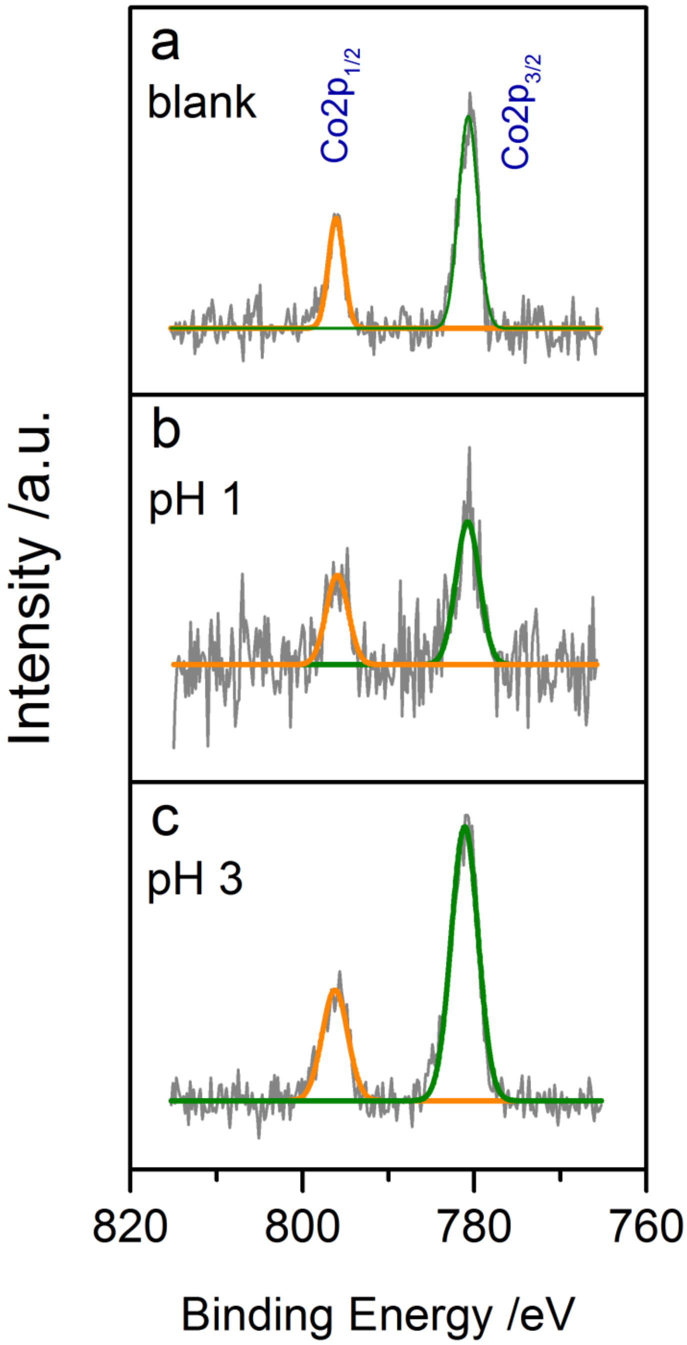

Supplementary Figure 10. XPS spectra of (a) freshly prepared cobalt protoporphyrin immobilized PG electrode; (b) cobalt protoporphyrin immobilized PG electrode after electrolysis at -1.5 V in 0.1 M $HClO_4$ solution (pH = 1) for 1 hour and (c) cobalt protoporphyrin immobilized PG electrode after electrolysis at -0.8 V in 99 mM $NaClO_4$ + 1 mM $HClO_4$ solution (pH = 3) for 1 hour. Figure (b) and (c) show that the CoPP remains intact on the electrode after electrolysis at very negative potentials. X-ray photoelectron spectroscopy (XPS) spectra were collected on a Quantera SXM (Scanning XPS microprobe) spectrometer equipped with Al Kα (1486.6 eV) X-ray source. The source was operated with a 25 W emission power, beam size of 200 µm and pass energy of 224 eV. The resolution of the spectrometer was 0.2eV and 0.8 eV for high resolution element scan and survey spectra, respectively.

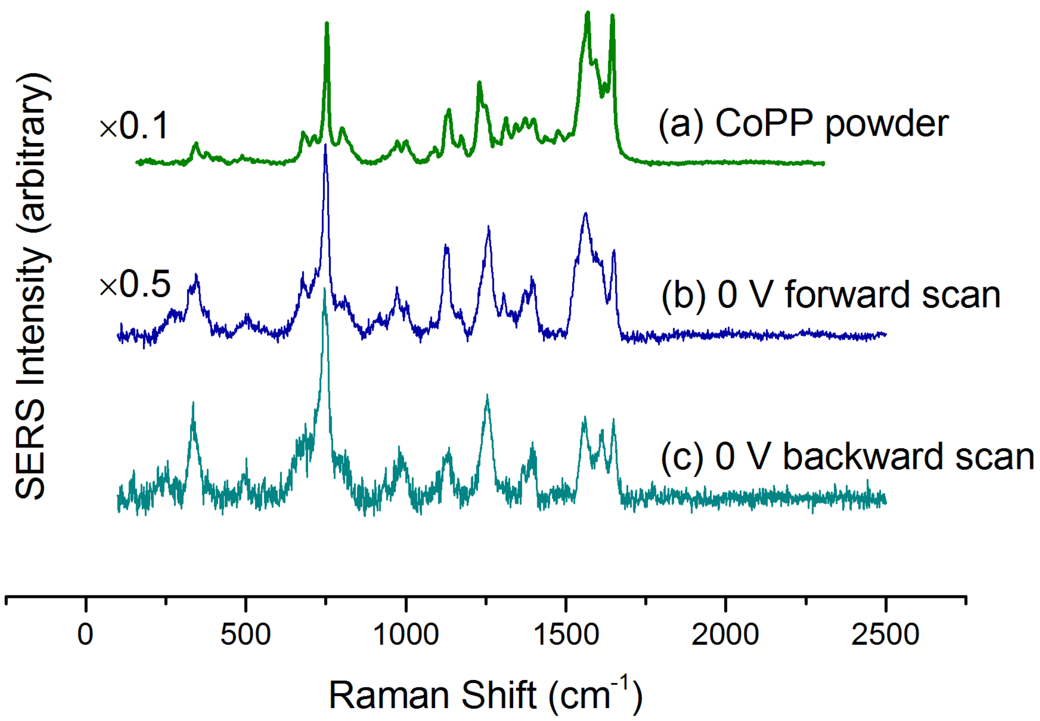

Supplementary Figure 11. SERS spectra of (a) cobalt protoporphyrin powder; (b) cobalt protoporphyrin immobilized Au electrode at 0 V before scanning and (c) cobalt protoporphyrin immobilized Au electrode at 0 V after voltammetry scanned to negative potentials. Au nano particles were synthesized as in reference 2, which were drop-cast onto a clean Au electrode. The electrode was then dipped into 0.5 mM cobalt protoporphyrin solution to saturate the surface with porphyrin. Cobalt protoporphyrin immobilized electrode was held at different potentials started from 0 V to –1.5 V vs RHE with 100 mV interval and then back while SERS spectra was collected at each potential.

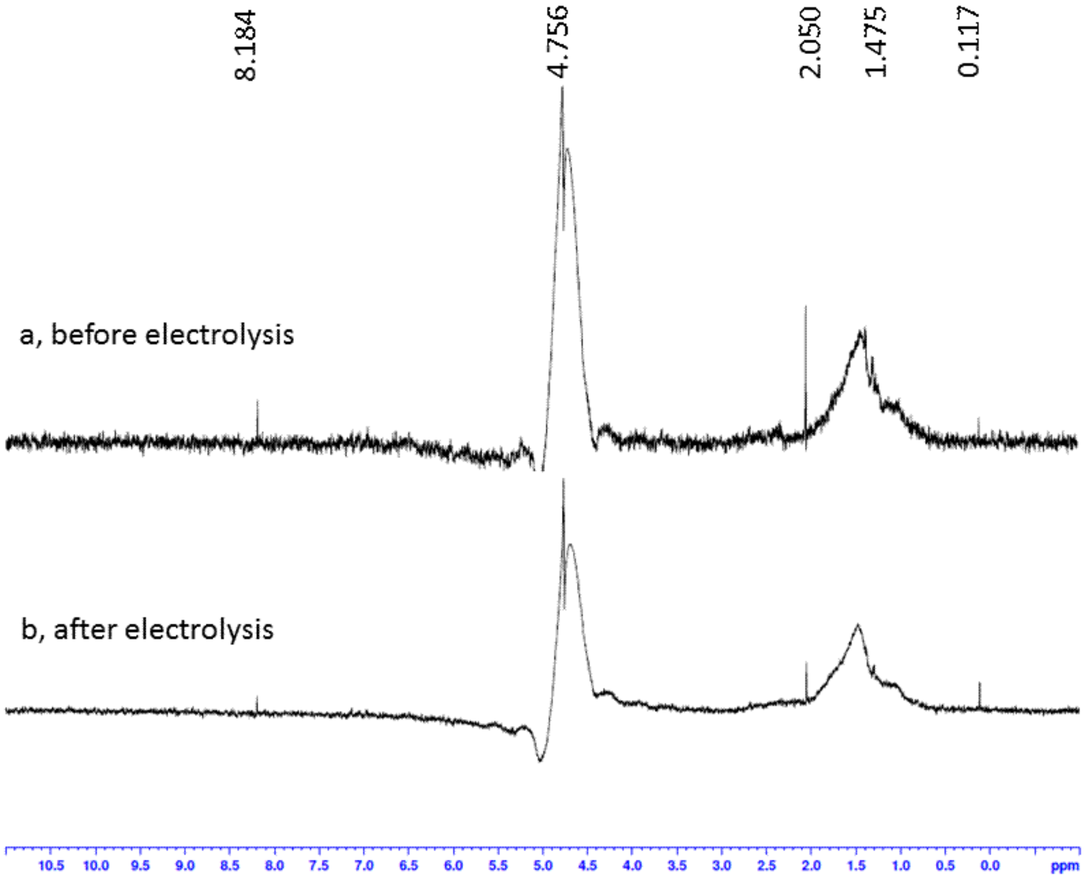

Supplementary Figure 12. NMR spectra of electrolyte (a) before and (b) after long-term electrolysis in CO_2 saturated 0.1 M $HClO_4$ solution on CoPP immobilized PG electrode. The electrolysis was conducted for 5 hours in a H-cell with 15 mL volume in each compartment. 100 uL solution in working electrode compartment was collected after electrolysis for NMR measurement.

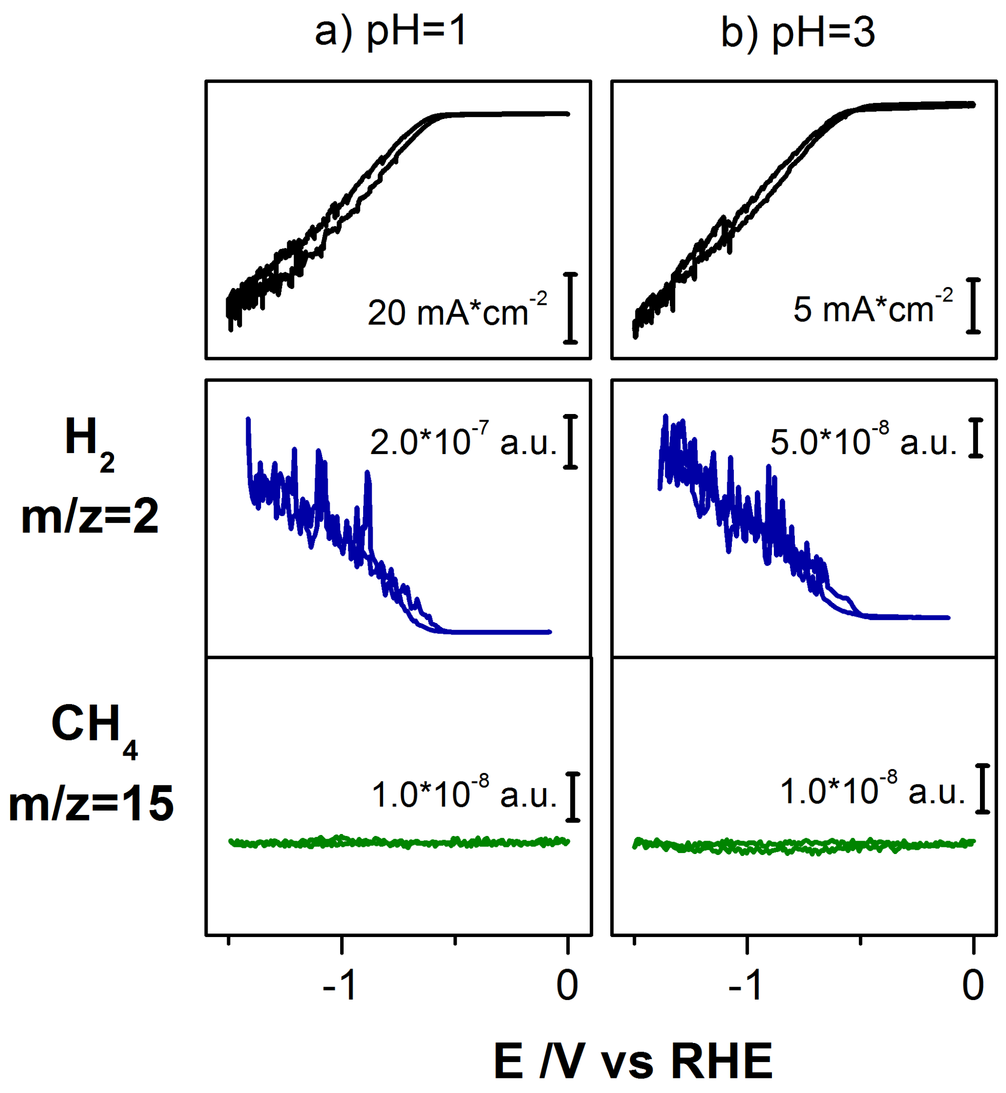

Supplementary Figure 13. Combined CV-OLEMS measurement of electrochemical reduction of formic acid on cobalt protoporphyrin modified pyrolytic graphite electrode, a) in 0.1 M $HClO_4$ (pH=1) solution and b) in 1 mM $HClO_4$ + 99 mM $NaClO_4$ (pH=3) solution, showing the cyclic voltammetry (upper panel) and the hydrogen and methane formation (bottom panels). This experiment demonstrates that formic acid was not reduced at either pH=1 or pH=3.

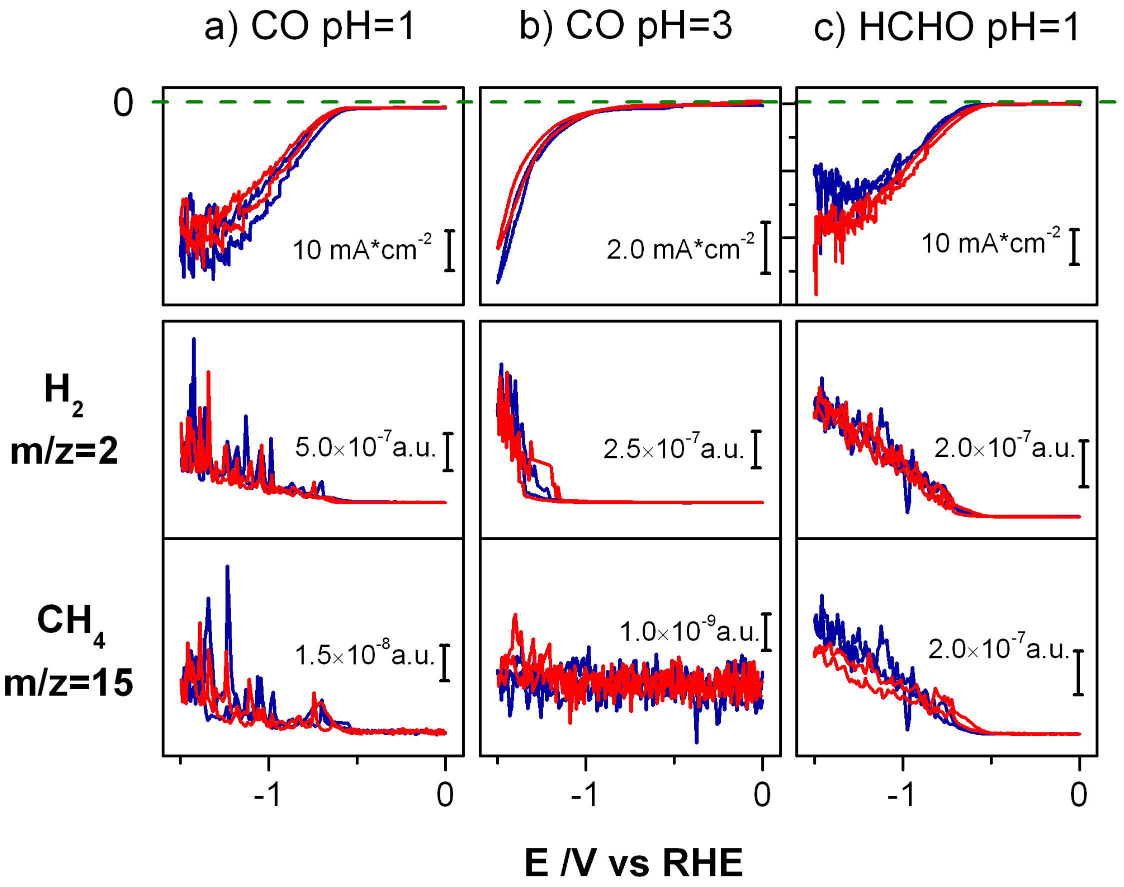

Supplementary Figure 14. Volatile products identification by OLEMS (showing unnormalized data) during electrochemical reduction of CO and HCHO. Cyclic voltammetry of CO reduction in (a) 100 mM $HClO_4$ and (b) 1mM $HClO_4$ + 99 mM $NaClO_4$ saturated with CO at first cycle (blue curve) and second cycle (red curve) with associated mass fragments of volatile products detected with OLEMS. (c) Cyclic voltammetry of HCHO (5 mM) reduction in 100 mM $HClO_4$ at first cycle (blue curve) and second cycle (red curve), with associated mass fragments measured with OLEMS. Scan rate: 1 mVs^{-1}.

Supplementary Table 1 Faradaic Efficiencies of Products from the electrochemical CO_2 reduction on cobalt protoporphyrin immobilized PG electrode at different pH and pressure. Electrolyte: 0.1 M perchlorate solution.

pH	Pressure	Potential vs RHE /V	Current mA	Faradaic Efficiency, % *					
				CH_4	CH_3OH **	CO	HCOOH ***	H_2 ****	total
3	1 atm	-0.6	-0.16	--	--	18.5±1.2	--		
		-0.8	-0.33	--	--	37.4±2.0	--		
		-1.0	-1.22	0.22±0.01	--	14.8±2.8	--		
		-1.2	-0.71	0.10±0.0	--	18.2±2.4	--		
		-1.4	-2.67	0.06±0.0	--	6.1±1.3	--	90.8±6.7	97.0±8.0
	10 atm	-0.6	-0.44	--	--	60.3±2.0	--		
		-0.8	-0.62	--	--	50±2.9	--		
1	1 atm	-1.2	-28.2	0.54±0.08	<<1%	0.6±0.1	<1%		
	10 atm	-0.8	-1.32	2.3±0.2	--	6.7±0.4	--		

* Error bars were determined from 3-8 samples taken every 6 minutes during a long-term electrolysis experiment after the system had reached steady state.

** Methanol was not detected using GC, but only as m/z=31 during OLEMS experiments. The Faradaic Efficiency towards this product must be (very) low, << 1%.

*** Formic acid formed near the surface of electrode was detected using HPLC. Faradaic efficiency was determined from the bulk concentration of formed formic acid, which was lower than the limitation of HPLC, 1%.

**** The sensitivity to H_2 in the GC is much lower than that of other gases; we give only the full Faradaic efficiency results for the high-current experiment at pH=1/p=1 atm.

Supplementary References

1. Palomar-Pardavé M, Scharifker BR, Arce EM, Romero-Romo M. Nucleation and diffusion-controlled growth of electroactive centers: Reduction of protons during cobalt electrodeposition. *Electrochim Acta* 2005, **50**(24)**:** 4736-4745.

2. Frens, G. Controlled Nucleation for the Regulation of the Particle Size in Monodisperse Gold Suspensions. *Nature Phys Sci* 1973**, 241**:20
